# Scapulothoracic and Glenohumeral Kinematics During Daily Tasks in Users of Manual Wheelchairs

**DOI:** 10.3389/fbioe.2015.00183

**Published:** 2015-11-20

**Authors:** Kristin D. Zhao, Meegan G. Van Straaten, Beth A. Cloud, Melissa M. Morrow, Kai-Nan An, Paula M. Ludewig

**Affiliations:** ^1^Rehabilitation Medicine Research Center, Department of Physical Medicine and Rehabilitation, Mayo Clinic, Rochester, MN, USA; ^2^Motion Analysis Laboratory, Division of Orthopedic Research, Mayo Clinic, Rochester, MN, USA; ^3^Department of Health Sciences Research, Mayo Clinic, Rochester, MN, USA; ^4^Biomechanics Laboratory, Division of Orthopedic Research, Mayo Clinic, Rochester, MN, USA; ^5^Program in Rehabilitation Science, Department of Physical Medicine and Rehabilitation, University of Minnesota, Minneapolis, MN, USA

**Keywords:** kinematics, shoulder pain, activities of daily living, manual wheelchair users, spinal cord injury

## Abstract

**Background:**

Rates of shoulder pain in individuals who use manual wheelchairs (MWCs) as their primary means of mobility have been reported to be as high as 70% during activities of daily living. Current prevailing thought is that mechanical impingement of the soft tissues that reside within the subacromial space between the humeral head and coracoacromial arch is a major contributor to the shoulder pain in users of MWCs. The subacromial space size is directly related to the kinematics at the shoulder joint. Yet to be answered are questions about which common daily tasks are characterized by the most potentially detrimental kinematics.

**Objective:**

The purpose of this analysis was to quantify and compare potentially detrimental kinematics in three common tasks performed by individuals with spinal cord injury and shoulder pain. These data will add to the body of knowledge and test common assumptions about relative risk of tasks.

**Design:**

A cross-sectional study of 15 MWC users with shoulder pain.

**Methods:**

Electromagnetic surface sensor measures of mean and peak scapulothoracic (ST) internal and downward rotation, anterior tilt, and glenohumeral (GH) internal rotation were compared across propulsion, weight relief, and scapular plane abduction tasks using one-way repeated-measure ANOVA.

**Results:**

Statistical differences were observed between the tasks for all rotations. Mean ST anterior tilt was greater in weight relief and propulsion than during scapular plane abduction (24°, 23°, and 13° of anterior tilt, respectively). Mean GH axial rotation during weight relief was more internally rotated than during propulsion and scapular plane abduction (9°, 26°, and 51° of external rotation, respectively).

**Limitations:**

Surface-based measures of kinematics are subject to skin motion artifact, especially in translation which was not addressed in this study.

**Conclusion:**

Each task presented with specific variables that might contribute to risk of developing shoulder “impingement” and pain. These data may assist therapists in their assessment of movement contributions to shoulder pain in this population, as well as in subsequent treatment planning.

## Introduction

Over 1.5 million persons in the United States use manual wheelchairs (MWCs) (Kaye et al., 2000), and ~20% are users of MWCs secondary to a traumatic or non-traumatic spinal cord injury (SCI) (Kaye et al., 2002). Users of MWCs with SCI are forced to perform many movements within the confines of the MWC. Therefore, they rely heavily on their shoulders as a means of locomotion, to perform weight relief lifts in order to avoid skin breakdown due to pressure on insensate skin, to reach for objects from a seated position, and to perform other activities of daily living. Thus, rates of shoulder pain in individuals with SCI have been reported to be as high as 70% during activities of daily living, negatively affecting their quality of life and independence (McCasland et al., 2006). Therefore, it is imperative that we better understand the secondary chronic conditions users of MWCs are confronted with, to preserve independence over the life span.

**Figure 1:**
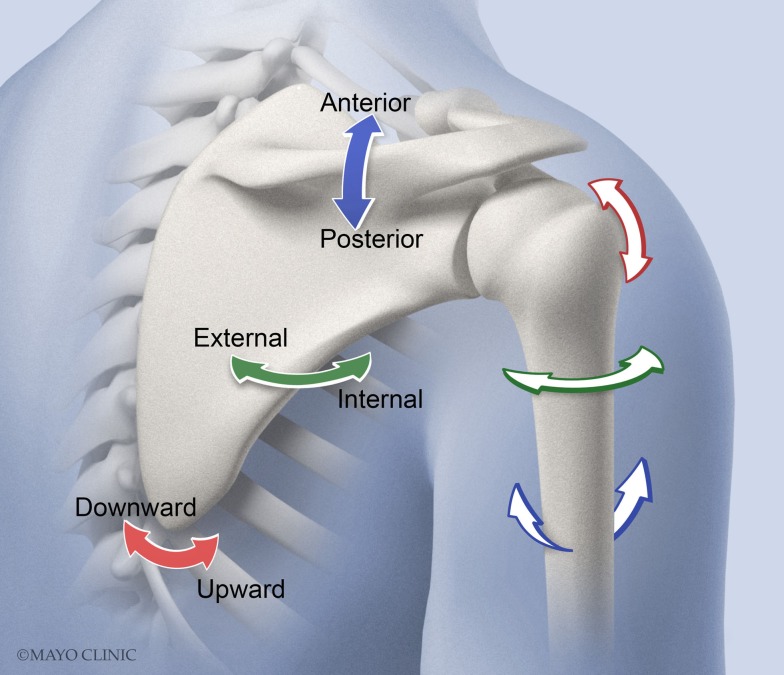
**Scapulothoracic (ST) rotation is comprised of internal/external rotation (solid green arrow), upward/downward rotation (solid red arrow), and posterior/anterior tilting (solid blue arrow)**. Glenohumeral (GH) motions of interest (not shown for clarity) include plane of elevation (anterior or posterior to the scapular plane) (blue outlined arrow), elevation (red outlined arrow), and axial rotation (internal/external) about the humeral long axis (green outlined arrow).

Current prevailing thought is that mechanical impingement of the soft tissues that reside within the subacromial space between the humeral head and coracoacromial arch (Dyson-Hudson and Kirshblum, 2004) is a major contributor to the shoulder pain in users of MWCs. The rotator cuff tendons, biceps tendon, and bursa reside within the subacromial space; therefore, reduction in this space, because of the tasks MWC users perform, provides a potential mechanism for injury or pain to the shoulder joint (Flatow et al., 1994; Schneeberger et al., 1998; Soslowsky et al., 2000). The subacromial space is defined and directly affected by the orientations of the humerus and scapula and the resulting rotation angles of the glenohumeral (GH) and scapulothoracic (ST) joints. Clinicians and biomechanists use slightly different language to describe the same movement, and this is important to keep in mind when reviewing kinematic literature. Biomechanists describe GH motions as: (1) humeral elevation, (2) motion in the horizontal plane during humeral elevation (anterior or posterior to the scapular plane), and (3) axial rotation (internal/external) about the humeral long axis (Figure 1). Clinicians describe these motions as: (1) flexion/extension/abduction/adduction, (2) horizontal abduction/adduction, and (3) internal/external rotation. Biomechanists describe ST motions as internal/external rotation, upward/downward rotation, and posterior/anterior tilting. The protraction/retraction terminology that clinicians use is a combination of scapular internal rotation and translation of the scapula on the thorax due to sternoclavicular joint protraction/retraction.

When comparing individuals with and without shoulder pain/subacromial impingement symptoms, kinematics have been shown to differ among able-bodied individuals during humeral elevation (Ludewig and Reynolds, 2009). Therefore, it is believed that altered kinematics may negatively impact the subacromial space. These potentially detrimental kinematics are as follows (compared with asymptomatic group values): (1) increased ST internal rotation, (2) increased ST downward rotation, (3) increased ST anterior tilt, and (4) increased GH internal rotation (Lukasiewicz et al., 1999; Ludewig and Cook, 2000; Endo et al., 2001). We believe that some of the tasks MWC users perform on a daily basis predispose them to these potentially detrimental kinematics, regardless of the presence or absence of pain.

A few investigations that include both ST and GH kinematics have been performed to characterize activities commonly performed by users of MWCs, and kinematics presumed to reduce the subacromial space were reported. Morrow et al. (2011) quantified ST and GH kinematics during level and ramp propulsion as well as during weight relief lifts in 12 users of MWCs using an optical-based motion capture system and a scapula tracker. Subjects tested were all pain free. At the point of peak shoulder loading, the weight relief lift displayed significantly less GH external rotation (or more relative GH internal rotation) than the level and ramp propulsion (16° and 19° differences, respectively). Riek et al. (2007) analyzed the shoulder kinematics of various activities of daily living in five individuals with SCI using an electromagnetic system and found that during the initial and maximum loading phases of weight relief, the humerus was significantly more internally rotated than in standing posture using a standing frame (15° and 38° differences, respectively). Nawoczenski et al. (2012) evaluated weight relief and transfer kinematics in individuals with and without shoulder pain. Throughout the transfer task, the group experiencing shoulder pain demonstrated increased anterior tilt of the trailing arm compared with the asymptomatic group. Overall, very little comparative data are available assessing the kinematics of common tasks theorized to contribute to subacromial impingement risk.

With limited data, the understanding of how kinematics during wheelchair-based tasks influence mechanical subacromial impingement and rotator cuff soft tissue compression has not matured. Yet to be answered are questions about which movement and/or environmental optimization interventions will have the biggest impact on maintenance of shoulder health. Yet, clinicians must make decisions on a daily basis as to how to intervene for MWC users who have developed shoulder pain. In order to test current clinical theories and assumptions regarding task-related development of shoulder pain in MWC users, further direct comparison of shoulder kinematics are needed in common wheelchair-based tasks. In particular, there are no direct comparisons of tasks presumed most detrimental, such as MWC propulsion and weight relief raise, to the kinematics of raising the arm while in a seated position. Based on the literature in able-bodied individuals (Kebaetse et al., 1999), the latter task may be of equivalent risk for development of shoulder subacromial impingement in MWC users functioning from a seated position.

GH and ST kinematics during level MWC propulsion, weight relief, and humeral elevation in the scapular plane were collected as part of a study investigating the effects of an exercise intervention for individuals experiencing mild-to-moderate amounts of shoulder pain during daily activities (Van Straaten et al., 2014). We sought to compare these three wheelchair-based tasks to each other ergonomically, rather than to compare between subjects with and without shoulder pain. Subjects with shoulder pain may differ from those without due to causative or compensatory kinematic changes (Nawoczenski et al., 2012; Lawrence et al., 2014). We sought a symptomatic group to not confound the analysis with both presence and absence of pain and to allow for the high likelihood and relevance of pain in this population. The purpose of this analysis was to quantify and compare potentially detrimental kinematics in three common tasks performed by individuals with SCI and shoulder pain. We hypothesized that differences existed in ST internal rotation, ST downward rotation, ST anterior tilt, and GH internal rotation between the tasks of MWC propulsion, weight relief, and humeral elevation in the scapular plane. These data will add to the body of knowledge and test common assumptions about relative risk of tasks.

## Materials and Methods

### Subject Population

Fifteen individuals who use MWCs as their primary means of mobility participated in the study. These subjects were recruited and signed written informed consent as part of a study approved by the Mayo Clinic Institutional Review Board, investigating shoulder kinematics as well as effects of an exercise intervention for shoulder pain. The kinematic data used for this analysis were recorded at the baseline time point, prior to intervention. Inclusion criteria were assessed by a licensed physical therapist and included: 18–65 years; SCI; shoulder pain during daily activities with clinical symptoms consistent with mechanical subacromial impingement (Park et al., 2005; Michener et al., 2009); use of a MWC as the primary means of mobility in the home and community; and ability to perform independent transfers and sit independently. Sixty-five years was selected as the upper limit for recruitment as we felt this represented the limit of fairly active MWC users whose results may not be confounded by age-related degenerative shoulder complaints. Exclusion criteria were cognitive impairments that limited the ability to independently follow instructions; pain deemed to be of cervical origin; presence of adhesive capsulitis (loss of >25% of range of motion in at least three directions; this included assessment of GH internal/external rotation with the arm at 90° abduction, as well as flexion, and abduction); significant injury to the tested shoulder in which pre-injury status was not attained; gross instability; or suspected labral tears. Finally, subjects were not included in the study if they had allergies to the adhesive tape that was used to attach the motion sensors.

### Subject Setup

**Figure 2:**
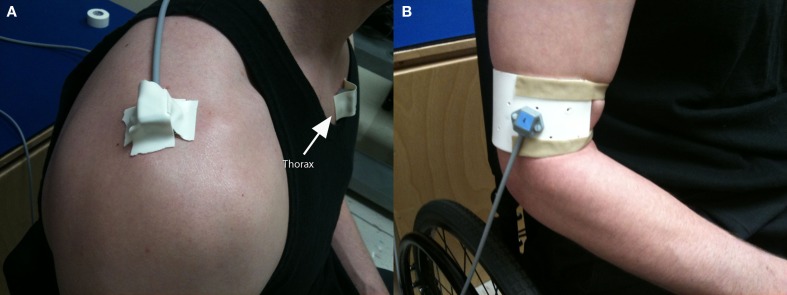
**Attachment of electromagnetic sensors on thorax and scapula (A), and distal humeral cuff (B)**.

The subjects were asked to sit in their own MWC in a comfortable seated posture while sensors were applied. Electromagnetic sensors (RX2, Polhemus, Inc.; Colchester, VT, USA) were attached to the painful limb via double-sided medical-grade adhesive tape to the sternum (just beneath the sternal notch), the acromion (on the superior surface of the posteromedial aspect of the acromion, at the junction with the scapular spine), and to the humerus (on the lateral side of a thermoplastic cuff secured to the distal humerus, just proximal to the epicondyles) (Karduna et al., 2001; Hamming et al., 2012). Care was taken to ensure that the acromial sensor was placed on the flat superior surface of the posteromedial aspect of the acromion, at the junction with the spine of the scapula. The arm was elevated prior to securing the sensor so that the sensor was placed medial to the muscle bulk of the deltoid. Thin medical tape was secured over the sensors and attached to ~0.5 cm of adjacent skin to minimize sensor movement (Figure 2). Data were collected at 240 Hz using MotionMonitor (Innovative Sports Training, Inc., Chicago, IL, USA) software, and a standard range transmitter (Polhemus, Inc., Colchester, VT, USA).

All subjects’ MWCs were tested for interference with the recording equipment and were deemed acceptable (i.e., the distance between two electromagnetic sensors rigidly attached to a wand in close proximity to the MWC remained constant). Each subject sat in his/her own MWC atop aluminum rollers with a resistance that was pre-determined to be similar to overground propulsion from pilot testing; the resistance of the rollers was not changed between the subjects. Local anatomical coordinate systems were defined on each body segment according to International Society of Biomechanics standards (X positive anterior, Y positive superior, Z positive to the right) (Wu et al., 2005). The shoulder joint center was defined using a functional (i.e., movement-based) approach (Meskers et al., 1999; Biryukova et al., 2000).

### Experimental Conditions

Before each activity, the movements were explained to the subjects and they were encouraged to become familiar with the experimental setup and practice the movements. Two movements, propulsion and weight relief, were closed-chain tasks that required a load through the hand; scapular plane abduction was an open-chain task, without a weight in the hand. The subjects were asked to perform two repetitions of scapular plane abduction, treadmill propulsion, and weight relief raises, without taking a break between repetitions of each task. However, between tasks, subjects were asked to take as much time as needed for a break period. The beginning position for scapular plane abduction trials was with the humerus next to the trunk, the elbow extended, and the palm of the hand facing forward so that the subjects’ ulnar side of the forearm or hand rested on their MWC rim. Then, they were asked to move their arm through 90° of abduction, at an angle oriented ~40° anterior to the coronal plane, and then reverse the movement in the same plane by adducting to the starting position. Plane of movement of 40° anterior to the coronal plane was verified using the MotionMonitor software at the time of data collection and adjusted if needed. Subjects were asked to perform the movement at a consistent speed, with verbal counting “up, two, three, four, down, two, three, four” by the investigator, returning to contact the hand rim between trials. For the weight relief movement, the individual was asked to start with his/her hands in their lap, and then after a verbal clue, move at a comfortable pace and place their hands on the MWC hand rim or wheel, lift their weight until their elbows were fully extended, and hold for 2 s before lowering their body weight; the second trial was performed identically. For propulsion, subjects were asked to start with their hands in their lap and then when verbally prompted to begin, proceed with placing their hands on the hand rim followed by at least two full propulsion movements in the style they normally use to propel.

Just prior to the data collection, subjects were asked to complete the Wheelchair Users Shoulder Pain Index (WUSPI) (Curtis et al., 1995a,b), a validated survey for assessing shoulder pain during daily activities. The WUSPI requires that participants rate their shoulder pain intensity on a 0–10 visual analog scale anchored by “no pain” and “worst pain ever experienced” during 15 common daily activities, and is rated for each activity over the preceding 7 days. A total score of zero indicates no pain, while 150 indicates severe pain.

### Data Analysis

Euler angles were generated to describe the position and orientation of the scapula relative to the thorax (ST), the humerus relative to the scapula (GH), and the humerus relative to the thorax (HT) at each frame across the times series. The International Society of Biomechanics standard definitions were used for the ST (YX′Z″) and HT (YX′Y″) rotations; however, an alternative sequence (Phadke et al., 2011) was used to describe GH rotations (XZ′Y″) to avoid singular positions near the neutral position and provide a more clinically interpretable rotation sequence.

**Figure 3:**
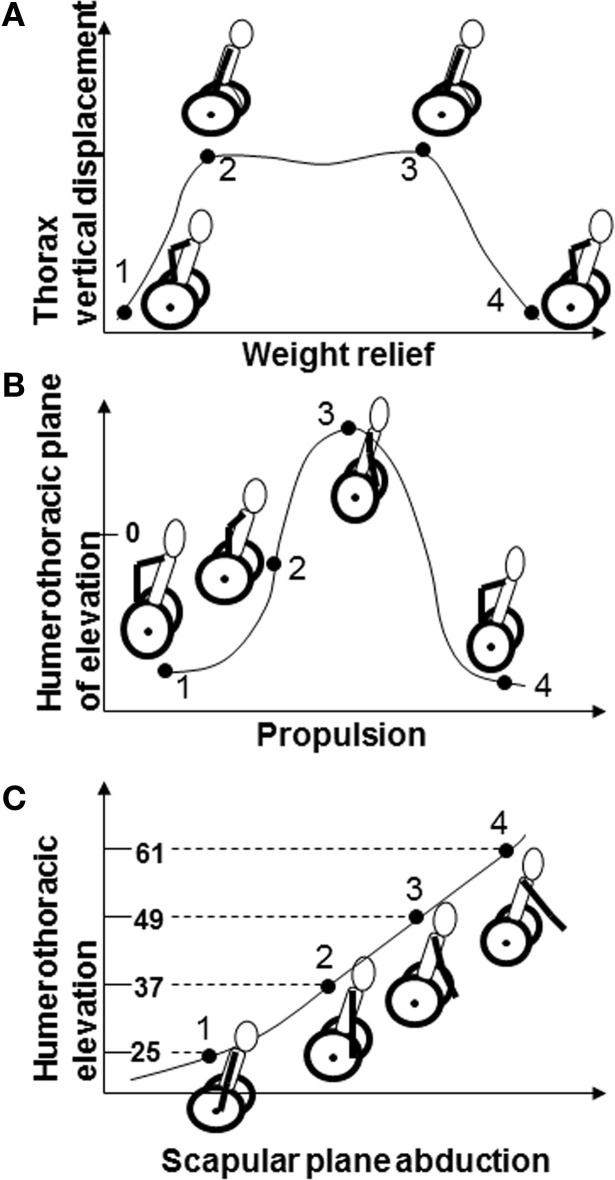
**Events 1–4 for (A) weight relief include initiation of lift, beginning of hold, end of hold, and end of lowering phases; (B) propulsion include beginning of push, mid-push, end of push, and end of recovery phases; (C) scapular plane abduction including 25°, 37°, 49°, and 61° of humerothoracic elevation**.

From the time series kinematics, four movement events were defined for each task so that they represented the overall excursion across the movements (Figure 3). The four events for weight relief were chosen interactively based on the vertical displacement of the trunk sensor. The start of the raise (event 1) was chosen as an upward vertical displacement. The start of the hold (event 2) was chosen as the end of the upward vertical displacement. The end of the hold (event 3) was chosen as the initiation of downward vertical movement of the trunk sensor. And, the return to the start position (event 4) was chosen as the end of downward vertical movement of the trunk. The four propulsion events were also selected interactively but were based on the HT plane of elevation rotation values. The start of propulsion (event 1) was chosen as the initiation of forward movement of the humerus. The point where the hand left the hand rim at the end of the push phase (event 3) was chosen as the end of forward movement of the humerus. Half way through the push phase (event 2) was calculated as half the time to event 3. The end of propulsion (event 4) was chosen as the end of backward movement of the humerus. This approach for identifying propulsion cycles has been validated against measurements of force applied on the push wheels during propulsion using a SMARTWheel (Three Rivers Holdings, LLC) (unpublished). There is a synchronous relationship between the application of force to the wheels and the HT plane of elevation when propelling on the rollers. Scapular plane abduction events were determined automatically based on the HT elevation values. The start of scapular plane abduction (event 1) was selected as the time at which 25° of elevation occurred. Events 2, 3, and 4 were defined as the times at which 37°, 49°, and 61° of elevation occurred, respectively. Twenty-five degrees was chosen as the minimum elevation value that all subjects could attain while seated in their MWC, 61° was chosen as the expected peak elevation for the other two tasks and was chosen so that all three tasks were taking place in similar regions of the movement space to prevent confounding of humeral elevation angle differences on task comparisons of kinematics. Peak kinematic values were also selected from the original time series curves for the kinematics in the direction of motion believed to be responsible for a reduction in the subacromial space (ST internal and downward rotation, ST anterior tilt, and GH internal rotation).

### Statistics

Descriptive analyses of the mean and standard deviations of the time series data for each of the ST and GH rotations were performed. Repeatability of trial event data for outcome variables, for all three movement tasks, were determined using intraclass correlation coefficients ICC(1,1) and standard errors of measurement (SEMs). Separate analyses were performed for the mean kinematic values and peak kinematic values for all dependent variables (the three ST rotations and one GH rotation believed to influence subacromial space). The normality of the data was determined prior to performing one-way repeated-measure ANOVAs with the task condition (propulsion, weight relief, and scapular plane abduction) as the within-subject factor. The data were checked for sphericity. If the Mauchly’s criterion was violated, the *p* values were corrected. Bonferroni *post hoc t*-tests were performed when a significant effect of task was found. All statistical analyses were performed using SAS Enterprise Guide 4.3 (Cary, NC, USA), and the significance level was set at 0.05. Potential covariates for this analysis included age, injury level, years of MWC use, and level of pain (WUSPI). Correlations were determined between these values and the dependent variables with a threshold of 0.5 for inclusion in the statistical model.

## Results

### Subject Population

Fifteen subjects (13 males and 2 females) were recruited according to the recruitment criteria for study participation (Table 1). Subjects had a mean age of 39 (SD 12) years, mean weight of 81 (SD 18) kg, averaged 14 (SD 9) years of MWC use, and presented with mild-to-moderate levels of pain [WUSPI 37.9 (SD 24.4)]. Subjects’ injury levels ranged from C6–C7 to L2 with one subject who was post-polio, presenting clinically as a lower lumbar SCI.

**Table 1:** **Subject demographics**.

Characteristic	Participants (*n* = 15, mean ± SD)
Age (years)	39 ± 12
Gender	13 males, 2 females
Weight (kg)	81 ± 18
Injury level (range)	C6/7 to L2, post-polio
Time using MWC (years)	14 ± 9

## Kinematics

The ICC values for the trial-to-trial event data ranged from 0.92 to 0.98 and SEMs ranged from 1.1° to 2.3° across the three tasks. As the data were relatively repeatable, the second trial was used for further analysis in the study so that the data represented actual movement kinematics rather than means across trials. None of the covariate regressions reached a correlation of 0.5 (the highest correlation was 0.15), so covariates were not included in subsequent analyses. Parametric statistics were used for all analyses as kinematic data were deemed to be normally distributed.

**Figure 4:**
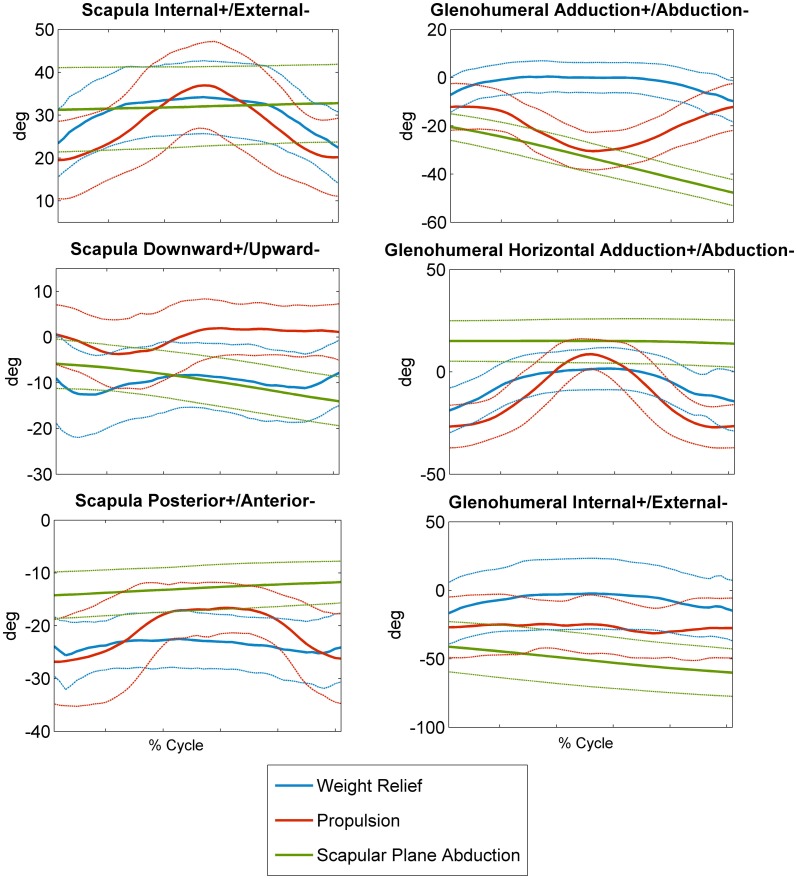
**Subjects’ mean (±1 SD, dashed lines) scapulothoracic (internal+/external−, downward+/upward−, posterior+/anterior−) and glenohumeral (lowering+/elevation−, horizontal adduction+/abduction−, internal+/external−) rotations for weight relief, propulsion, and scapular plane abduction movements**.

**Figure 5:**
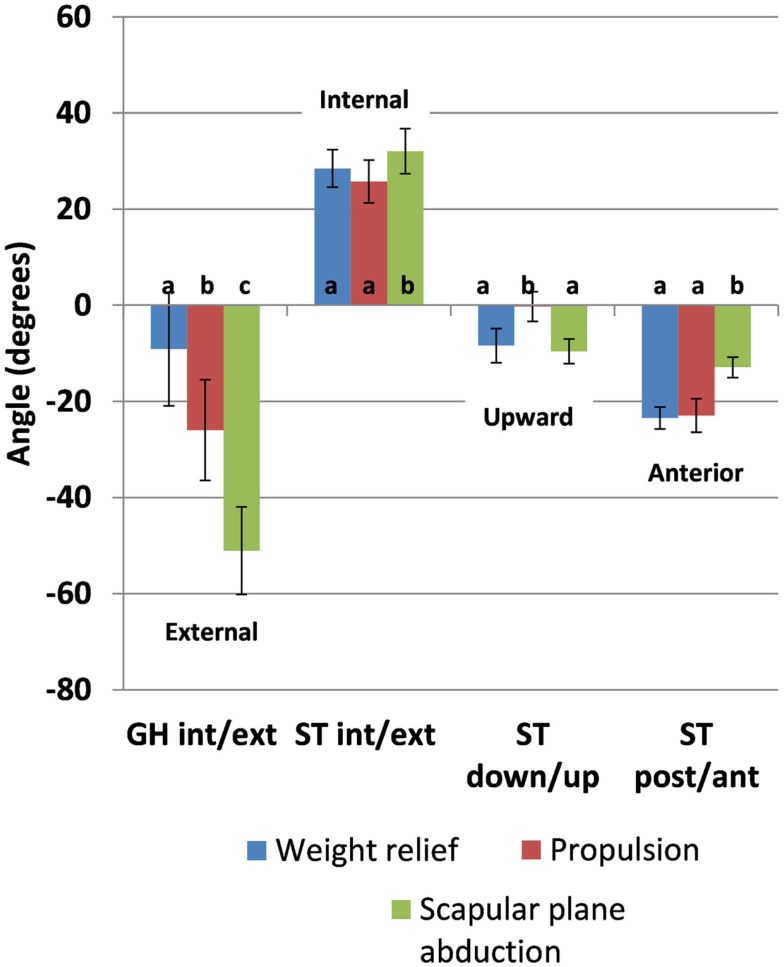
**Kinematic means and 95% confidence interval values for between-task comparison of the mean for the three tasks**. Letters indicate significance (*p* < 0.05); the same letter indicates no significant difference between values (GH, glenohumeral; ST, scapulothoracic; int, internal; ext, external; down, downward; up, upward; post, posterior; ant, anterior).

Mean time series data across all subjects for each ST and GH rotation and each task are depicted (Figure 4). There were significant effects of task on event *means* of all kinematics of interest: GH axial rotation (*F*(2,28) = 121.42, *p* < 0.0001), ST internal rotation (*F*(2,28) = 11.14, *p* = 0.0003), upward rotation (*F*(2,28) = 22.79, *p* < 0.0001), and tilt (*F*(2,28) = 51.03, *p* < 0.0001). *Post hoc* testing demonstrated that mean GH axial rotation during weight relief was more internally rotated than during propulsion and scapular plane abduction (9°, 26°, and 51° of external rotation, respectively). Scapular plane abduction had greater mean ST internal rotation than both weight relief and propulsion tasks (32° versus 26° and 29° of ST internal rotation respectively, Figure 5). ST downward rotations were greater during propulsion (3° upward rotation) than weight relief (8° upward rotation) and scapular plane abduction (10° upward rotation, Figure 5). No differences were found between scapular plane abduction and weight relief in *post hoc* testing. Mean ST anterior tilt was greater in weight relief and propulsion than during scapular plane abduction (24°, 23°, and 13° of anterior tilt, respectively, Figure 5). Weight relief and propulsion were not significantly different with respect to mean ST anterior tilt (Figure 5).

**Figure 6:**
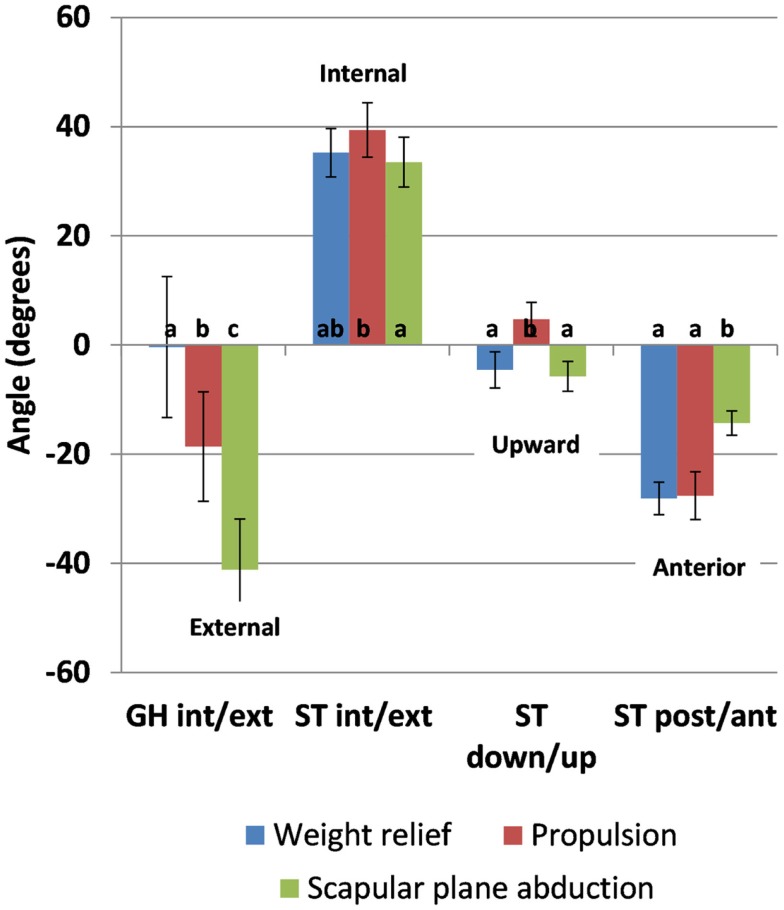
**Peak kinematic values in GH internal rotation (-0.4°, -18.7°, and -41.2°), ST internal rotation (35.2°, 39.4°, and 33.5°), downward rotation (-4.6°, 4.7°, and -5.7°), and anterior tilt (-28.1°, -27.6°, and -14.3°) and 95% confidence interval values for between-task comparison for the weight relief, propulsion, and scapular plane abduction, respectively**. Letters indicate significance (*p* < 0.05); the same letter indicates no significant difference between values (GH, glenohumeral; ST, scapulothoracic; int, internal; ext, external; down, downward; up, upward; post, posterior; ant, anterior).

Results from the one-way repeated-measure ANOVA analysis of the *peak* kinematic values show similar trends to the event *means* for all kinematics measures except ST internal rotation; peak values reached in each of the rotations during each task can be found in Figure 6. Maximum GH internal rotation was greater during weight relief than propulsion and scapular plane abduction [*F*(2,28) = 75.44, *p* < 0.0001]. Propulsion resulted in greater peak ST internal rotation (39°) than scapular plane abduction (34°); it did not significantly differ from weight relief values (35°) [*F*(2,28) = 4.07, *p* = 0.0282]. Peak ST downward rotation was greater during propulsion (5° downward) than during weight relief (5° upward) and scapular plane abduction (6° upward) [*F*(2,28) = 25.06, *p* < 0.0001]. Peak anterior tilt during weight relief (28°) was greater than during scapular plane abduction (14°), but not significantly different than during propulsion (28°) [*F*(2,28) = 40.83, *p* < 0.0001].

## Discussion

Currently, the tasks most problematic for MWC users in the development of shoulder pain are often assumed to be MWC propulsion, weight relief raises, and transfers. However, this assumption has never directly been tested, and many other activities, such as arm elevation from a seated position, have the potential to be equally detrimental. Our investigation sought to begin to test these assumptions. Due to our working assumption that a reduction in the subacromial space is related to development of shoulder pain, we have chosen to investigate the kinematics of these movements as a more direct indication of the space, and not the kinetics that led to the ensuing motions. This study compared ST and GH kinematics believed to reduce the subacromial space between level propulsion, a weight relief maneuver, and an arm raise in the scapular plane. Not surprisingly, there were significant differences in the kinematics of different tasks. The meaningful part of these comparisons lie in the interpretation of the magnitude of these differences as well as the value that these findings will bring to the topic when combined with more direct measurements of subacromial space. The kinematics observed in this study provide some of the evidence needed to prioritize detrimental motions and tasks warranting further investigation.

In addition to determining that kinematics are different, one method of interpreting the potential importance of magnitudes of differences between the kinematic tasks is to qualitatively assess the differences from a neutral hanging posture. Neutral hanging arm posture has been defined in healthy subjects in a standing posture using bone-pin mounted sensors (Ludewig et al., 2009). It is known that in the hanging arm position, the soft tissue structures that reside within the space have adequate clearance, so deviations away from the hanging arm position (for both ST and GH articulations) in certain directions are thought to be detrimental. In the ISB coordinate system used in this study, the natural hanging posture is ~29° of ST internal rotation, 5° of ST downward rotation, 10° of ST anterior tilt, and 2° of GH external rotation (Ludewig et al., 2009).

Mean event data revealed a slightly greater overall ST internal rotation during scapular plane abduction than during the other two movements, with differences ranging from 3° to 6°. While these differences are statistically significant, we believe the differences to be small and likely do not have a substantial impact on the underlying subacromial space. Further, the values are very similar to the neutral hanging arm posture in healthy subjects. Therefore, we do not believe our ST internal rotation data are concerning for the MWC user population during these three tasks. ST upward/downward rotation differed by only 7° between tasks and, on average, the scapula remained in an upwardly rotated position. However, the peak rotation during propulsion reached a downward rotated position, which may place users at risk during this task given the elevated position of the humerus. Given the nature of the reduced upward rotation data during propulsion, therapists planning interventions for shoulder pain in MWC users should consider an assessment of an individual’s upward rotation during propulsion. If reduced upward rotation is present, exercises to improve lower serratus anterior (primary scapular upward rotator) activation and function during propulsion may be important to consider in treatment planning.

ST anterior tilt and GH internal rotations exhibited larger differences between tasks and deviations from the neutral hanging arm position were greater for anterior tilt. Mean ST anterior tilt was greater in weight relief and propulsion than during scapular plane abduction by up to 11° and also represented deviations away from the hanging arm position, while scapular plane abduction did not (Figure 5). GH internal rotation values during weight relief were significantly greater, but did not deviate substantially from the neutral hanging arm posture. Based on current assumptions regarding relationships between angular kinematics and the subacromial space, weight relief and propulsion tasks warrant further investigation and more direct measurements of subacromial space. These study results suggest potentially greater focus on improving upward rotation angles during propulsion tasks, and reducing anterior tilt angles during weight relief raise. The data also suggest that the ST muscle groups deserve attention in rehabilitation interventions.

ST and GH time series data during weight relief were similar in ranges and values to previous data obtained from individuals with spinal cord injury without presence of pain (Morrow et al., 2011) except for ST anterior tilt values (52°–55° versus 20°–28° of anterior tilt in the current study). The anterior tilt differences are likely attributed to the use of a scapular tracker (versus an acromial sensor) for tracking scapula motion in the Morrow et al. investigation. A scapula tracker is most sensitive to error in the anterior/posterior tilt rotation values due the nature of its attachment to the scapular spine. Further, subject-specific coordinate systems were not assigned for each subject in the Morrow study. A transformation based on cadaveric data was used to reference the scapula tracker motion to the anatomical coordinate system of the scapula. As such, our data for anterior tilt cannot be directly compared to the Morrow et al. investigation.

When comparing to previously published data in able-bodied subjects during propulsion (Lu et al., 2002), differences in ST and GH internal rotation ranges (ST: 12°–29° versus 19°–39° of internal rotation in the current study, GH: 5°–10° internal rotation versus 19°–35° of external rotation in the current study) may be attributed to the study populations. Due to variable innervation of the trunk musculature in our spinal cord injured subjects, we have noted that subjects often have a more kyphotic posture and forward head position. This posture would encourage ST internal rotation, which would in turn cause GH rotation to be more externally rotated given a similar humeral position. ST rotations during propulsion in a spinal cord injured population (Morrow et al., 2011) had similar internal/external rotation values (33°–40° of external rotation) to the current study.

Comparisons of our data were made to previous scapular plane abduction studies in able-bodied subjects that included time series data from 25° to 61° of HT elevation (McClure et al., 2001; Ludewig et al., 2009) from a standing position. Ranges of rotations were similar between the previous work and the current study; however, in the current study, the scapula was in greater internal rotation [35°–38° (McClure et al., 2001; Ludewig et al., 2009) versus 45° of internal rotation in the current study] and anterior tilt [7°–12° of anterior tilt (McClure et al., 2001; Ludewig et al., 2009), and 2°–4° of posterior tilt (McClure et al., 2001) versus 16°–18° of anterior tilt in the current study]. These findings can potentially be attributed to the seated posture of users of MWCs who may lack postural stability and may have greater spinal kyphosis.

There are limited studies reporting on the kinematics of activities of daily living in users of MWCs, and none that we are aware of interpreting the findings as they relate to a potential reduction in the subacromial space. Therefore, there are limited relevant studies to compare to. While results of this study do not directly test a clinical intervention, we feel these findings will add important information to the literature that will help guide clinical decision-making of therapists addressing shoulder movement disorders in this population, as well as informing future research.

It is important to note that this investigation was not a comparison between painful and pain-free subjects, but rather a comparisons of the tasks themselves in MWC users with shoulder pain. We believe that the constraints of performing these tasks from a MWC likely have greater impact on kinematics than the presence or absence of pain. This presumption is supported by the lack of substantial association of the kinematics of subjects with their WUSPI scores. Pain also does not have a clear and consistent outcome with regard to affecting movement patterns (Ludewig and Cook, 2000; Nawoczenski et al., 2012). Subjects with shoulder pain have demonstrated disparate results as compared to controls, demonstrating both increases and decreases in key variables such as ST upward rotation. These disparate results have been suggested to represent both causation and compensation. Some subjects may alter kinematics in a compensatory pattern in response to pain, while others may have altered kinematics leading to pain. In addition, our analysis focused only on presumed kinematic mechanisms of subacromial rotator cuff compression, which occurs at lower arm elevation angles. Future work is intended to investigate tasks in this population at higher angles of elevation, where mechanical internal impingement may occur.

### Limitations

When interpreting our study findings, it is important to understand its limitations. Surface markers are known to have error as compared to bone-mounted sensors or imaging techniques for recording shoulder kinematics. However, comparison of bone-mounted and skin sensor data have shown errors in humerus and scapula skin sensor acquisitions of ≤4° during shoulder movements <60° of humeral elevation (Karduna et al., 2001; Ludewig et al., 2002), which is the range we investigated. This investigation focused on the effect of rotational changes during the tasks rather than including the humeral head translation values.

Additionally, this study focused specifically on kinematics thought to be detrimental for subacromial space. Through kinematic results, we can begin to infer which tasks may be putting the shoulder in disadvantageous positions. However, further investigation is warranted to understand the forces acting on the upper extremity and focused evaluations of the subacromial space during MWC-based tasks. An additional limitation is that task kinematics were collected in the same order for all subjects, so it is possible that an order effect existed in the data. No subjects complained of fatigue, and subjects were allowed a break between tasks if desired. It is also possible that study outcomes for the scapular plane abduction may have differed if the subjects had lifted a hand-held weight; however, this seems unlikely based on previous research (de Groot et al., 1999). Further, each subject sat in his/her MWC atop aluminum rollers with a resistance that was not changed between subjects (i.e., not personalized to subject weight). In addition, all subjects underwent a clinical exam by a physical therapist to ensure appropriate innervation and function of their shoulder musculature. While it is possible that the two subjects with higher level spinal cord injuries had some undetected differences in shoulder muscle innervation, it is unlikely.

## Conclusion

Current assumptions regarding relationships between angular kinematics and the subacromial space implicate greater amounts of ST internal rotation, downward rotation, anterior tilt, and GH internal rotation as disadvantageous. Based on these assumptions and the overall kinematics observed in this study of the tasks evaluated, weight relief and propulsion seem to be most detrimental for the shoulder. However, each task presented with specific variables that might contribute to risk of developing shoulder “impingement” and pain. These data may assist therapists in their assessment of movement contributions to shoulder pain in this population, as well as in subsequent treatment planning. This data add to our understanding of the relative risks associated with various MWC-based activities.

## Conflict of Interest Statement

The authors declare that the research was conducted in the absence of any commercial or financial relationships that could be construed as a potential conflict of interest.
